# Joanne Knight Breast Health Cohort at Siteman Cancer Center

**DOI:** 10.1007/s10552-022-01554-1

**Published:** 2022-01-21

**Authors:** Graham A. Colditz, Debbie L. Bennett, Jennifer Tappenden, Courtney Beers, Nicole Ackermann, Ningying Wu, Jingqin Luo, Sarah Humble, Erin Linnenbringer, Kia Davis, Shu Jiang, Adetunji T. Toriola

**Affiliations:** Division and Public Health Sciences and Siteman Cancer Center, Washington University School of Medicine, 660 South Euclid Avenue, Campus Box 8100, Saint Louis, MO 63110 USA

**Keywords:** Prospective, Cohort, Women, Biomarkers, Mammography, Benign breast disease

## Abstract

**Purpose:**

The Joanne Knight Breast Health Cohort was established to link breast cancer risk factors, mammographic breast density, benign breast biopsies and associated tissue markers, and blood markers in a diverse population of women undergoing routine mammographic screening to study risk factors and validate models for breast cancer risk prediction.

**Methods:**

Women were recruited from November 2008 to April 2012 through the mammography service at the Joanne Knight Breast Health Center at Washington University in St. Louis, Missouri. Baseline questionnaire risk factors, blood, and screening mammograms were collected from 12,153 women. Of these, 1,672 were excluded for prior history of any cancer (except non-melanoma skin) or diagnosis of breast cancer within 6 months of blood draw/registration for the study, for a total of 10,481 women. Follow-up is through linking to electronic health records, tumor registry, and death register. Routine screening mammograms are collected every 1–2 years and incident benign breast biopsies and cancers are identified through record linkage to pathology and tumor registries. Formal fixed tissue samples are retrieved and stored for analysis. County-level measures of structural inequality were derived from publicly available resources.

**Results:**

Cohort Composition: median age at entry was 54.8 years and 26.7% are African American. Through 2020, 74% of participants have had a medical center visit within the past year and 80% within the past 2 years representing an average of 9.7 person-years of follow-up from date of blood draw per participant. 9,997 women are continuing in follow-up. Data collected at baseline include breast cancer risk factors, plasma and white blood cells, and mammograms prior to baseline, at baseline, and during follow-up.

**Conclusion:**

This cohort assembled and followed in a routine mammography screening and care setting that serves a diverse population of women in the St. Louis region now provides opportunities to integrate study of questionnaire measures, plasma and DNA markers, benign and malignant tissue markers, and repeated breast image features into prospective evaluation for breast cancer etiology and outcomes.

## Background

Many of the current cohorts of women for cancer study in the USA are insufficient for examining factors from the biology to the environment that are associated with breast cancer risk among a diverse group of women. Most of these ongoing cohorts of women for the study of cancer have extensive questionnaire risk factor data collected for all participants, yet they are made up predominantly of White women. Smaller subcohorts have blood samples for prospective analysis of hormones, metabolic markers, and DNA [1]. These cohorts typically have mammograms retrieved on only a small subset of the participants, if at all [2, 3]. For example, the Mayo Mammography Health Study includes 19,924 women seen at Mayo Clinic mammography service from 2003 to 2006 and includes breast cancer risk factor measures and follow-up through electronic health records, but lacks racial diversity [4]. Further, despite growing evidence that mammographic breast density and additional markers of parenchymal texture [5] are strong risk factors for breast cancer [6–8], few studies integrate repeated mammography measures [9] in addition to questionnaire risk factors and blood-based markers. Even fewer cohorts routinely integrate breast tissue from benign and malignant lesions [1, 10, 11]. To address gaps in race/ethnic composition of cohorts, newer studies such as the Black Women’s Health Study [12] have been established, yet these have similar challenges in assembling tissue samples and image data. This study has, however, provided valuable insights to risk prediction in Black women [13]. Recent validation of mammography-based breast cancer risk model based on AI included 7 data sets relying heavily on Emory for mammograms from African American women [14]. Thus, a resource gap exists limiting epidemiologic investigations and validation of risk prediction models. As Potter noted over 15 years ago [1], the integration of all these data sources is essential to fully capitalize on genomics, proteomics, geographic and environmental measures, and tissue to integrate data on host and tumor phenotype. While he proposed a million-person “last cohort” we here describe baseline data on a cohort that meets many of the principles he outlined.

## Purpose of the study

Dr. Colditz and colleagues established the Joanne Knight Breast Health Cohort at Siteman Cancer Center to collect, store, and ultimately share comprehensive data sets and tissue specimens for future research. The screening mammography service at Siteman and Washington University School of Medicine offered us the potential to recruit a diverse population of women [15] and to bring routine mammography images and all breast biopsies into the cohort follow-up as a feature of the prospective data collection. This thus fills two of the major gaps in existing US cohort studies and facilitates study of risk factors and validation of models for breast cancer risk prediction.

The Joanne Knight Breast Health Center at Siteman Cancer Center at Washington University School of Medicine, St. Louis, Missouri provides mammography services for women from varying socioeconomic and racial backgrounds in the St. Louis region, including those with coverage through the Missouri breast and cervical cancer screening programs (Centers for Disease Control and Prevention and state funded), the Komen Fund and Barnard Fund coverage for the uninsured, and regularly insured women with private insurance or Medicare coverage. All women are screened with the same technology (Hologic). The mammography service stores all images and as of 2019 all screening used tomosynthesis (Hologic).

## Materials and methods

### Recruitment

Posters describing the study were placed in waiting areas and women attending mammographic breast screening or diagnostic procedures at the Joanne Knight Breast Health Center were approached to participate, all of them completing extended data collection for breast cancer risk estimation. The Joanne Knight Breast Health Center screens approximately 25,000 women and does high risk and diagnostic screening for another 15,000 women per year [15]. Women aged 18 and older attending the Breast Health Center were eligible to enroll. More than 50% of eligible women attending for screening mammograms chose to enroll. Males were excluded, as were women with self-reported blood transfusion within the past 4 months, and self-reported HIV +, Hepatitis B, or C +.

The variables needed for the simplified Rosner–Colditz breast cancer risk prediction model [16] (these include age, menopausal status, age at menopause, pregnancy history, history of benign breast disease, and current menopausal hormone therapy (estrogen alone, estrogen plus progestin, progesterone alone, and other), current BMI, height, and daily alcohol intake, see measures below) have been routinely collected since 2010 and risk estimates are incorporated into reporting from breast health screening mammograms. Those invited to the study and agreeing were consented and then proceeded to blood draw. 20 mls of blood were drawn and aliquoted for storage at −80 °C in the Siteman Tissue Procurement Core liquid nitrogen freezer system. Aliquots of white blood cells and plasma are stored separately in cryotubes.

Cohort participants consented to (1) retrospective and prospective review of medical records (including radiologic images, pathology reports); (2) one-time 20 ml blood draw; (3) access to tissue not required for clinical care (e.g., breast biopsy tissue following conclusive clinical pathology assays); and (4) optional future contact for the purposes of long-term follow-up and/or to recruit for other related research projects. Record linkage identifies new mammograms, biopsies, and other visits to BJC Health Care facilities. BJC is a non-profit health care organization serving metro St. Louis, mid-Missouri, and Southern Illinois.

Enrollment from November 2008 to April 2012 included 12,153 women who provided blood and risk factor data. A survey of 158 women who opted not to enroll over a two-week period in October 2009 showed most women who did not participate cited a lack of time to give the blood sample (30.4%). The next largest group (19.0%) wanted more time to think about participation. The remaining reasons for not participating included not wanting to give a blood sample (8.9%), not wanting researchers to have access to their medical records (8.9%), and (13.9%) provided no answer. The majority of the women came in for screening mammogram and a subset for diagnostic follow-up (5.4% of total cohort). Of these enrolled women, 1,672 had a history of cancer at enrollment leaving 10,481 women free from cancer at baseline.

### Methods of follow-up

Follow-up of cohort participants as determined by mammography and other clinic visits through December 2020 was 78% seen in 2019 or 2020; a further 4.4% seen most recently in 2018 and a further 2.4% in 2017. All women remain under surveillance for return to follow-up mammography. Follow-up is passive through medical record linkages every 6 months, annual tumor registry searches, and annual mortality searches. This results in over 80% active follow-up for women seen within the last 36 months. The average person-years of follow-up through most recent contact is 9.2 person-years.

### Exposures measures

At enrollment a baseline questionnaire, blood draw, and mammogram were obtained along with address for follow-up and for geocoding for measures of structural inequality. Baseline blood samples were taken and stored in multiple aliquots; DNA extraction (3 aliquots of 1 ml) and plasma aliquots of 1 ml (6 per participant) and placed into cryovials and stored at − 80 °C in LN_2_ freezers.

Women self-reported breast cancer risk factors on entry to the cohort. These are drawn from established and validated measures [17]. The baseline questionnaire assessed height, weight at age 18, current weight and weight at menopause, age at menarche, age at first birth, age at each subsequent birth, parity, menses ceased (yes/no), age at menopause and surgical removal of uterus, with removal of ovaries or without removal of ovaries, and age at hysterectomy; family history of breast cancer (mother and/or sister), Ashkenazi Jewish heritage; history of benign breast biopsy; current use of hormone therapy (yes / no and type of hormone therapy, including duration), current use of oral contraceptives (yes / no) and duration, current alcohol intake, current smoking status, and cigarettes per day.

Mammograms: a screening mammogram 12–24 months prior to baseline, at baseline, and subsequent follow-up screening have been identified and stored. These images are stored along with BI-RADS density report recorded (*a* = almost entirely fat, *b* = scattered areas of fibroglandular density, *c* = heterogeneously dense, *d* = extremely dense). Routine screening mammograms were obtained using Hologic machines.

County-level measures of structural inequality: We summarize multiple measures of county-level structural inequality that were included for relevance to population health and health disparities. First, we include five multi-dimensional factors representing several domains of structural inequality. Each factor consists of four or five variables clustering around the following themes: racial and economic segregation; population change; opportunity for socioeconomic advancement; economic environment; and population and housing characteristics. They were derived using exploratory factor analysis (EFA) in SAS 9.4 (SAS Institute Inc., Cary, NC) and theory-driven choices. The data were publicly available and previously compiled by the Health Inequality Project [18]. We also include three versions of the Index of Concentration at the Extremes (ICE) for (1) race, (2) income, and (3) income and race combined. The ICE measures compare the most advantaged groups to the least advantaged groups and the combined ICE measure compares higher-income White or Caucasian populations to lower-income Black or African American populations. These measures describe the distribution of extreme privilege and deprivation for these indicators across a specified area [19]. Finally, we include measures of area-level debt delinquency for any debt and for medical debt since area-level indebtedness has been shown to impact household finances as well as available neighborhood-level services [20] which have implications for neighborhood stability and subsequent health. The measures of area-level debt delinquency are publicly available through the Urban Institute [21]. All variables were appended to participant’s geocoded county of residence at the time of enrollment for a total of 224 unique counties.

## Results

The cohort free from cancer at baseline includes 10,481 women. The distribution by race/ethnicity reflects the racial distribution in our catchment area and is summarized in Table 1. Almost 27% of the cohort is Black or African American, less than 1% are Asian, and 69% are White or Caucasian. Of these women, 1% identify as Hispanic. Women were aged 30 to 94 at entry with 90% between ages 35 and 69 at blood draw and median age 54. Furthermore, 4.3% of participants come from rural residential addresses defined by RUCA codes.

**Table 1:** Race and ethnicity and age distribution of women participating in the Joanne Knight Breast Health Cohort at Siteman Cancer Center, Washington University

	Ethnicity			Total
	Hispanic or Latina	Not Hispanic or Latina	Not reported	
American Indian or Alaska native	4 (0.04%)	16 (0.2%)	1 (0.01%)	21 (0.2%)
Asian	2 (0.02%)	76 (0.7%)	9 (0.09%)	87 (0.8%)
Black or African American	4 (0.04%)	2556 (24.4%)	227 (2.2%)	2797 (26.7%)
White	73 (0.7%)	6719 (64.1%)	489 (4.7%)	7281 (69.5%)
Multiracial	2 (0.02%)	55 (0.5%)	7 (0.07%)	64 (0.6%)
Not reported	23 (0.2%)	71 (0.7%)	137 (1.3%)	231 (2.2%)
Total	108 (1.0%)	9493 (90.6%)	880 (8.4%)	10,481 (100%)

Age at entry	*n* (%)	African American * n* (%)	White N (%)	All other races * n* (%)
< 35	65 (0.6%)	17 (0.2%)	45 (0.4%)	3 (0.03%)
35–39	191 (1.8%)	51 (0.5%)	130 (1.2%)	10 (0.09%)
40–44	1297 (12.4%)	397 (3.8%)	837 (8%)	63 (0.6%)
45–49	1691 (16.1%)	487 (4.6%)	1131 (10.8%)	73 (0.7%)
50–54	2041 (19.5%)	593 (5.7%)	1365 (13%)	83 (0.8%)
55–59	1848 (17.6%)	446 (4.3%)	1345 (12.8%)	57 (0.5%)
60–64	1492 (14.2%)	366 (3.5%)	1075 (10.3%)	51 (0.5%)
65–69	890 (8.5%)	195 (1.9%)	660 (6.3%)	35 (0.3%)
70–74	499 (4.8%)	128 (1.2%)	355 (3.2%)	16 (0.2%)
75–79	281 (2.7%)	72 (0.7%)	199 (1.9%)	10 (0.09%)
80+	186 (1.8%)	45 (0.4%)	139 (1.3%)	2 (0.02%)
Total	10,481	2797 (26.7%)	7281 (69.4%)	403 (3.8%)

Breast cancer risk factors at baseline are summarized in Table 2. Briefly, women were on average 54.8 years at enrollment and nulliparity was more common among White (20.4%) vs Black (11.5%) women. 61% of participants were postmenopausal at entry to the cohort. The mean body mass index (BMI) was 29.3 kg/m^2^ and of note it was higher for Black women (32.8 kg/m^2^) vs White women (28.0 kg/m^2^). During follow-up linkages to cancer registry and pathology records have identified 272 incident invasive breast cancers and 116 in situ lesions through October 2020. A total of 623 benign biopsy samples from 6/28/2010 through 12/31/2020 have been identified and are stored for centralized pathology review and classification. Through January 2021, we have confirmed 329 deaths within this cohort.

**Table 2:** Joanne Knight Breast Health Cohort selected characteristics at entry, 10,092 women free from cancer

Characteristics at recruitment	Total	African American	White	All other races
Number of women	10,481	2797 (26.7%)	7281 (69.5%)	403 (3.8%)
Year of birth, median (IQR)	1955 (± 14)	1956 (± 13)	1954 (± 14)	1957 (± 15)
Age at recruitment, years, median (IQR)	54.8 (± 13.9)	53.5 (± 13.5)	55.5 (± 13.9)	53.2 (± 14.9)
Nulliparous	1873 (17.9%)	321 (11.5%)	1486 (20.4%)	66 (16.4%)
Number of children, in parous women (mean, SD)	2.4 (± 1.2)	2.6 (± 1.5)	2.3 (± 1.0)	2.3 (± 1.1)
Menopausal—ceased menses	6395 (61%)	1707 (61.0%)	4462 (61.3%)	226 (56.1%)
Current smoker	1127 (10.8%)	550 (19.7%)	537 (7.4%)	40 (9.9%)
Does not drink alcohol	3547 (33.8%)	1374 (49.1%)	2013 (27.6%)	160 (39.7%)
Height in inches, mean, SD	64.5 (± 2.7)	64.3 (± 2.8)	64.6 (± 2.6)	63.7 (± 2.7)
Weight in pounds at baseline, mean, SD	173.6 (± 44.4)	193.2 (± 47.1)	166.6 (± 40.9)	164.3 (± 44.8)
Weight in pounds at 18, mean, SD	126.4 (± 25.3)	130.2 (± 30.0)	125.4 (± 23.6)	118.9 (± 19.6)
Body mass index, mean, SD	29.3 (± 7.3)	32.8 (± 7.6)	28.0 (± 6.6)	28.3 (± 7.3)
Add debt or other social determinants here				
BI-RADS Density baseline mammogram				
(a) Almost entirely fat	1075 (10.3)%	409 (14.6%)	631 (8.7%)	a8.7%)
(b) Scattered areas of fibroglandular density	5267 (50.3)%	1581 (56.5%)	3502 (48.1%)	a5.7%)
(c) Heterogeneously dense	3511 (33.5%)	684 (24.5%)	2683 (36.8%)	a5.7%)
(d) Extremely dense	452 (4.3%)	58 (2.1%)	362 (4.9%)	a7.9%)
(e) Not recorded	176 (1.7%)	65 (2.3%)	103 (1.4%)	8 (2.0%)
Follow-up				
To date				
Deaths	329	122 (4.4%)	197 (2.7%)	10 (2.5%)
Incident invasive breast cancers	270	56 (2.0%)	209 (2.9%)	5 (1.2%)
Incident in situ breast cancer	118	21 (0.7%)	94 (1.3%)	3 (0.7%)
Additional mammograms through Sept 2017, mean, SD	5.1 (± 2.7)	4.8 (± 2.6)	5.3 (± 2.7)	4.5 (± 2.5)
Benign biopsy tissues samples (6/28/2010–12/31/2020)	623(5.9%)	148 (5.3%)	442 (6.1%)	33 (8.2%)

Socioeconomic status varied among participants. 45.6% were living in counties with debt of any kind at or above 30% of the population (Table 3).

**Table 3:** Joanne Knight breast health cohort baseline—county-level structural inequality (*n* = 10,481 women, *n* = 224 counties)

Area level measure—county level	Number of counties represented	Number of women with non-missing value	Mean (SD)	Median (Min—Max)
Structural inequality factors				
Racial and economic segregation	219	10,243	1.55 (1.15)	1.11 (− 5.95 – 5.81)
Population change	219	10,243	− 0.52 (0.53)	− 0.61 (− 3.50 – 5.50)
Generational dispossession	219	10,243	1.37 (1.46)	0.71 (− 4.37 – 5.91)
Economic environment	219	10,243	− 0.90 (0.57)	− 0.91 (− 4.45 – 5.70)
Population and housing	219	10,243	0.73 (0.87)	1.00 (− 1.75, 25.31)
Index of concentration at the extremes (ICE)				
Income	224	10,248	− 0.06 (0.15)	− 0.03 (− 0.40 – 0.47)
Race	224	10,248	0.41 (0.33)	0.45 (− 0.35 – 0.99)
Income and race	224	10,248	0.07 (0.14)	0.16 (− 0.14 – 0.45)
			*n* (%)	
Debt delinquency				
Proportion of women living in counties with **debt of any kind** at or above 30%	224	10,248	4669 (45.6%)	
Proportion of women living in counties with **medical** debt at or above 30%	224	10,248	203 (2.0%)	

### Early findings

Plasma samples from the cohort have been evaluated for carotenoid concentrations and risk of proliferative benign breast disease diagnosed from baseline through April 2016 [22]. Among women under age 50 we observed that African Americans had lower levels of alpha and beta-carotene and higher levels of beta-cryptoxanthin and lutein/zeaxanthin. There was a suggested inverse association between plasma carotenoids and risk of proliferative benign breast disease. Ongoing analysis aims use this cohort to externally validate the Rosner–Colditz breast cancer risk model that includes mammographic breast density, breast cancer questionnaire risk factors, and polygenic risk scores [16]. The study also motivates novel statistical methods for breast image data analysis in the time to event setting [23–25]. For example, using supervised Functional Principal Component Analysis of baseline full-field mammographic images we reported methods [23] and refinement to accommodate the irregular boundary of the mammographic image [24].

## Conclusion

This new cohort brings breast images and pathology from routine care in a clinical setting that serves a diverse population into prospective epidemiologic investigations for breast cancer. The integration of blood markers in addition to questionnaire-based risk factors and tissue samples for all breast biopsies, in addition to repeated mammograms on participants, brings unique strengths to this cohort. Furthermore, the diversity of this population that is approximately one-quarter African American fills gaps in both breast cancer etiologies, risk prediction development, and validation of breast cancer risk models in diverse populations.

Although repeated visits to the breast health center for screening mammography could facilitate updated or repeated blood measures, the epidemiologic evidence and resources to justify this have not been assembled to date. However, because breast images are the product of the mammography visit, improving approaches to maximize use of the information in these repeated images appears to be the most efficient approach to improve risk stratification as part of routine breast health services.

## Data access

Through IRB approval of deidentified data, plasma or tissue samples can be shared with investigators. Applications submitted to Dr. Colditz are reviewed by an internal Siteman committee, including breast pathology, mammography, and Tissue Procurement Core leadership. Material Transfer agreements are developed once access is approved and data, tissue samples, or blood samples are shipped as agreed. The overall study is approved by the institutional review board at Washington University in St. Louis.

## References

[1] Potter JD (2004). Toward the last cohort. Cancer Epidemiol Biomark Prev.

[2] Byrne C, Colditz GA, Willett WC, Speizer FE, Pollak M, Hankinson SE (2000). Plasma insulin-like growth factor (IGF) I, IGF-binding protein 3, and mammographic density. Cancer Res.

[3] Oh H, Rice MS, Warner ET, Bertrand KA, Fowler EE, Eliassen AH (2020). Early-life and adult anthropometrics in relation to mammographic image intensity variation in the nurses' health studies. Cancer Epidemiol Biomark Prev.

[4] Olson JE, Sellers TA, Scott CG, Schueler BA, Brandt KR, Serie DJ (2012). The influence of mammogram acquisition on the mammographic density and breast cancer association in the Mayo Mammography Health Study cohort. Breast Cancer Res.

[5] Gastounioti A, Conant EF, Kontos D (2016). Beyond breast density: a review on the advancing role of parenchymal texture analysis in breast cancer risk assessment. Breast Cancer Res.

[6] Eriksson M, Czene K, Pawitan Y, Leifland K, Darabi H, Hall P (2017). A clinical model for identifying the short-term risk of breast cancer. Breast Cancer Res.

[7] Nguyen TL, Aung YK, Li S, Trinh NH, Evans CF, Baglietto L (2018). Predicting interval and screen-detected breast cancers from mammographic density defined by different brightness thresholds. Breast Cancer Res.

[8] Anandarajah A, Chen Y, Colditz GA, Hardi A, Stoll C, Jiang S (2021) Studies of parenchymal texture added to mammographic breast density and risk of breast cancer: a systematic review of the methods used in the literature. medRxiv. 11.16.21266374. 10.1186/s13058-022-01600-5 PMC9805242 36585732

[9] Anandarajah A, Chen Y, Stoll C, Hardi A, Jiang S, Colditz GA (2021) Use of repeated mammograms to evaluate risk of breast cancer: a systematic review of methods used in the literature. MedRxiv. 10.1101/2021.11.10.21266200

[10] Colditz GA (2010). Ensuring long-term sustainability of existing cohorts remains the highest priority to inform cancer prevention and control. Cancer Causes Control.

[11] Boffetta P, Colditz GA, Potter JD, Kolonel L, Robson PJ, Malekzadeh R (2011). Cohorts and consortia conference: a summary report (Banff, Canada, June 17–19, 2009). Cancer Causes Control.

[12] Rosenberg L, Adams-Campbell L, Palmer JR (1972). The Black Women's Health Study: a follow-up study for causes and preventions of illness. J Am Med Womens Assoc.

[13] Palmer JR, Zirpoli G, Bertrand KA, Battaglia T, Bernstein L, Ambrosone CB (2021). A validated risk prediction model for breast cancer in US Black Women. J Clin Oncol.

[14] Yala A, Mikhael PG, Strand F, Lin G, Satuluru S, Kim T, et al (2021) Multi-institutional validation of a mammography-based breast cancer risk model. J Clin Oncol JCO2101337. 10.1200/JCO.21.01337 PMC9148689 34767469

[15] Moore JX, Han Y, Appleton C, Colditz G, Toriola AT (2020). Determinants of mammographic breast density by race among a large screening population. JNCI Cancer Spectr..

[16] Rosner B, Tamimi RM, Kraft P, Gao C, Mu Y, Scott C (2021). Simplified breast risk tool integrating questionnaire risk factors, mammographic density, and polygenic risk score: development and validation. Cancer Epidemiol Biomarkers Prev.

[17] Colditz GA, Hankinson SE (2005). The Nurses' Health Study: lifestyle and health among women. Nat Rev Cancer.

[18] Chetty R, Stepner M, Abraham S, Lin S, Scuderi B, Turner N (2016). The association between income and life expectancy in the United States, 2001–2014. JAMA.

[19] Krieger N, Waterman PD, Spasojevic J, Li W, Maduro G, Van Wye G (2016). Public health monitoring of privilege and deprivation with the index of concentration at the extremes. Am J Public Health.

[20] Walks A (2013). Mapping the urban debtscape: the geography of household debt in Canadian cities. Urban Geogr.

[21] Braga B, McKernan SM, Quakenbush C. Debt in America: an interactive dashboard. Urban Institute, Washington, DC (2019). https://apps.urban.org/features/debt-interactive-map/.

[22] Cohen K, Liu Y, Luo J, Appleton CM, Colditz GA (2017). Plasma carotenoids and the risk of premalignant breast disease in women aged 50 and younger: a nested case-control study. Breast Cancer Res Treat.

[23] Jiang S, Cao J, Rosner B, Colditz GA (2021) Supervised two-dimensional functional principal component analysis with time-to-event outcome on mammogram imaging data. Biometrics. 10.1111/biom.13611 10.1111/biom.13611 PMC9160217 34854477

[24] Jiang S, Cao J, Colditz GA, Rosner B (2021) Predicting the onset of breast cancer using mammogram imaging data with irregular boundary. Biostatistics. 10.1093/biostatistics/kxab032 10.1093/biostatistics/kxab032 PMC10102887 34435196

[25] Jiang S, Colditz GA (2021) Extracting features from mammograms in addition to breast density improves risk prediction for breast cancer: preliminary application. Soc Epidemiol Res. https://epiresearch.org/wp-content/uploads/2021/06/2021-Abstract-Book-Final.pdf

